# The Recognition of Incipient General Paresis

**Published:** 1888-01

**Authors:** Ogden Backus

**Affiliations:** Rochester, N. Y., Late Assistant Physician State Lunatic Asylum, Utica, N. Y.


THE BUFFALO Medical and Surgical Journal

VOL. XXVII. JANUARY, 1888. No. 6

### Original Communications.

### THE RECOGNITION OF INCIPIENT GENERAL PARESIS.

By OGDEN BACKUS, M. D., of Rochester, N. Y.,

Late Assistant Physician State Lunatic Asylum, Utica, N. Y.

General paresis, or general paralysis of the insane, is a disease which rarely comes under the care and observation of the general practitioner, except in its early stages, and is then often not recognized, or is mistaken for some other form of insanity or disease of the nervous system. During a period of over four years at the State Lunatic Asylum, at Utica, there was a large number of general paretics admitted, and in the majority of cases the disease was unrecognized by the medical men who had made the necessary examinations and certificates. Dr. Gray, in an article read before the State Medical Society some years ago, said, that in the entire number of cases placed in the asylum since his connection with the institution, but few were recognized by the physicians in attendance before admission, and that most of the cases were brought by friends, in the confident expectation of a speedy recovery, based generally on the opinion of the family physician.

Dr. McDonald, formerly of the Ward's Island Asylum, states that an examination of the certificates sent by gentlemen in private practice to that asylum, shows that they recognized the true character of the malady in but three cases out of the thirty-five in which they made affidavit. Corroborative evidence of the want of familiarity with the early symptoms of the disease is often met with in the newspapers. They record the illness of some prominent individual, with the detail of such symptoms as point strongly to general paresis, and often conclude with the announcement that, "in the opinion of the family physician, a few weeks' rest will restore the patient to his usual health." The case of the celebrated actor, John McCullough, who died a few years ago in Philadelphia, is an example. For months, he was permitted to wander about the country, indulging in visionary schemes and the excesses peculiar to this disease. The newspapers frequently chronicled his vagaries, but steps were not taken for his comfortable and safe maintenance, until a large part of his fortune had been squandered. More recently, a famous author was admitted to an asylum with general paresis, but not until he had brought financial ruin on himself and family, and was only a wreck of his former self.

That a malady so grave, destroying not only mind, but life itself, so important in its medico-legal aspects, should thus escape the diagnostic acumen of a large number of medical men, is a matter of more than ordinary interest, and yet the symptoms are often so obscure, and masked under such a variety of forms, that physicians even resident in asylums for the insane, are often chary of giving a positive diagnosis until the case has been under observation for a period.

General paresis remained unrecognized until fifty or sixty years ago, and the first published notice of this disease in America, was by Dr. Luther Bell, Superintendent of the McLean Asylum, in his annual report for 1843, where he described a few cases. At present, a considerable proportion of those admitted to every asylum are of this malady. In an analysis of eleven hundred and ninety cases of insanity admitted to the State

Lunatic Asylum, at Utica, I found that ninety-one were patients suffering from this disease, or seven and six-tenths per cent. of all persons admitted. In the Ward's Island Asylum, the percentage is somewhat larger, doubtless due to the fact that it draws the insane from the more densely-populated cities, where the exciting causes of this disease are more prevalent than in the rural districts of Central New York. Dr. McDonald, in an analysis of sixteen hundred cases admitted to the above asylum, classifies two hundred and five as paretics, or about thirteen per cent. of the whole number admitted.

In order to recognize general paresis in its incipiency, a familiarity with its ætiology is almost indispensable. First among the predisposing causes is sex; a characteristic observed is its selection of the male in a large proportion; indeed, in the early history of the disease, writers upon this subject were inclined to regard it as appertaining to men only. Sankey gives the average liability of the sexes to general paresis in England, as five men to one woman. McDonald finds that the statistics of the two public asylums of New York city, for three years, compare as follows: Asylum for females, seven paretics admitted in a total of thirteen hundred and thirty-five cases. In the asylum for males, one hundred and sixty-six were general paretics, out of twelve hundred and thirty-eight cases admitted during the same period. In other words, insanity has taken the form of general paresis in but one-half of one per cent. in the females, while it is found in twelve and six-tenths per cent. of the males admitted to these institutions. By these figures, we also see that twenty-four men to one woman was the ratio, a statement which does not concur with my own observation. At the Utica Asylum, in 1883, there were thirty male and four female paretics admitted; in 1884, there were twenty-seven male and five female paretics admitted; while in 1885, there were admitted twenty men and five women suffering with this disease. These figures show that in Central New York, at least, the proportion is six males to one female.

Age is another predisposing cause, for general paresis is usually a disease of middle life. From Dr. Mickle's calculations, based on the thirty-second report of the Commissioners in Lunacy, I learn that the relative age of patients is forty-four per cent. between forty and fifty years, and thirteen per cent. between thirty and forty years. In the Ward's Island Asylum, of a hundred and forty cases admitted during a given period, sixty-six were between the ages of thirty-five and forty-five; thirty were between twenty-five and thirty-five; and forty were between forty-five and fifty-five; only four were under twenty-five, the youngest being twenty-three. The youngest case I have met with was twenty-seven years of age, and I believe the disease to be rare under thirty years.

The age from thirty-five to fifty years is the period in which general paresis is most commonly developed, for, as Mickle most beautifully describes, "this is especially the age of ambition, pride, selfishness, of speculation, of daring attempts on the heights of fame, of wealth, power, prestige and social position—the age of excessive and protracted intellectual labor—of anxious and strenuous efforts to provide for and ensure future success to a growing and exigent family—the age in which excessive physical labor is often undertaken, which, like intellectual labor, may be often sustained by too liberal potations, and which is no longer counteracted by the elastic power of accommodation of youth, or by its restfulness after fatigue. Then, too, it is the age most liable to chagrins, and mortifications of spirit, to losses, to disappointments—the age occasionally of sudden beggary after a life of toil and hard-earned success—the age at which so often the mirage of hope vanishes and its aerial castles dissolve; at which the projects of life fail, and crumble away, and its fire dies out. Then, also, it is too often that the spirit of the man wanders forlorn amidst the desolate groves of his affection, the desecrated temples of his noblest aspirations. No wonder that causes such as these acting upon those whose nervous systems have lost the elasticity of youth, whose blood and blood-vessels are further impaired by the effects of alcohol, whose naturally hyperæsthetic brains have been exhausted by irritable reaction to every strong impression—no wonder, I say, that in them, causes such as these should bring about a sudden or protracted hyperæmia—a slow, irritative form of degeneration, or even inflammation in the supreme centres of the organ of the mind."

Heredity may be an important factor, but, as Verga states, the hereditary affinities of general paresis are not with ordinary insanity, but with paralysis, apoplexy, cerebral-congestion and other brain diseases. Nevertheless, in a certain proportion of cases, I have found insanity to exist in one or the other of the family branches.

The causation of general paresis is a subject on which a variety of opinions are held. "The things that most excite and, at the same time, most exhaust the highest brain energy, are those that tend most strongly to cause this disease." Town, rather than rural life; a sanguine temperament; constant worry and irritation or intense intellectual work. Intemperance appears in every statistical table as a predominating cause of general paresis, and is often associated with exhaustive, heavy, physical labor or sexual excess. There is, however, a great difference of opinion in regard to sexual excess as a prime cause of general paresis. Dr. Maudsley lays great stress on it, and chiefly that which is carried on systematically by faithful married persons; but Dr. Mickle, on the contrary, says: "Having for years sought, and usually in vain, for a history of sexual excesses in my own cases, I do not hold with the view that excessive frequency of sexual intercourse is, by far, the most fertile cause of general paralysis. No doubt, in some cases, and particularly among the newly-married, this may be the cause; in others, one of the several causes of the disease. But I venture to submit that it is erroneous to pay an almost exclusive attention to this cause, as has been done by some authorities on the subject, certain of whom have gone so far as to assert their belief that when not due to excessive sexual intercourse, general paralysis owns another form of sexual evil, namely, masturbation, as its exciting cause, as if forsooth the life, both marital and non-marital, of men was but as an orgy of satyrs, either consumed with secret lust, or fitly partnered in salacious revelry with Bacchantes, lascivious of eye and wanton of limb."

In my own experience with the cases in asylum life, sexual excess seems rather to have been one of the prodromic symptoms, and not a cause *per se* of the disease. In an analysis of fifty-six cases, I attribute the disease in twenty-six to excessive and prolonged mental strain and overwork, supported by alcoholic stimulants; in sixteen, to intemperance and irregular habits; in four, to syphilis; in one, to sunstroke; in the remaining seven, the history furnished was so meagre as to render an opinion as to its causation little more than a guess.

In order to recognize general paresis in its incipiency, careful inquiry should be made into the habits of the individual, the temperament, family history, age, etc., all of which will greatly aid in diagnosis. The symptoms, especially in the early stage, are somewhat vague; the mode of commencement is generally gradual, although the disease may begin suddenly, an epileptiform seizure being the initial warning to the patient or to his friends; or motor symptoms may be the first observed; some slight embarrassment or hesitancy of speech is noticed, especially if under excitement; aural tinnitus, flushing and heat of face and head, due to vaso-motor disturbance. If, however, the intellectual powers be involved, a change occurs in the disposition and habits of the individual. He becomes forgetful, is inattentive, his business transactions are ill-judged, he may be moody, dull, apprehensive, suspicious, irritable, full of hypochondriacal fancies, his memory begins to fail, especially for recent events, and he is unable to apply himself thoroughly to any work. It may be that his sexual appetite is increased; and not infrequently, in taking the history of these cases, the wife of the individual will admit that for some time previous to suspicion of disease on the part of friends, her husband's sexual desire has been almost insatiable.

More commonly, instead of depression, there exists an expansive, speculative frame of mind. He is exalted, active, busy, full of chimerical schemes, elated, easily excited, unnaturally loud in conversation ; many of his friends, indeed, believe that he has taken a " wee drop too much," for the change in manner is the same which an extra glass of whiskey produces. Although naturally reticent, he becomes loquacious, confidential, buttonholes people on the street, relates matters of a private nature about his family, their prospects, what he intends to do for them, etc. ; is sleepless, although he does not complain of insomnia. His appetite is good, and he digests his food well. Perversion of the moral sense is one of the most important of the prodromic symptoms, especially from a medico-legal point of view. For some time, there may have been unwonted acts of indelicacy or impropriety. He may be led into all kinds of folly or crime, may make extraordinary purchases, commit thefts in the most open manner, and yet, aside from these changes, there is no absolute loss of intellect ; he may become a little duller, less initiative ; perhaps, to the casual observer, appearing in his normal condition, but a characteristic feature is, with but few exceptions, that all these changes occur unnoticed by the patient. He affirms that he is healthier than ever before, and any insinuation to the contrary serves only to irritate him. In one case, the first symptom shown of this perversion of the moral sense, was the fact of the patient's deliberately rising from the dining-room table, urinating in the corner of the room, and returning to his seat, as if nothing had occurred, much to the astonishment of those present.

The following case is given by Folsom, in Pepper's System of Medicine, vol. 5, p. 200: " A gentleman once committed an offence characteristic of general paralysis, in marrying a pretty servant girl while temporarily away from his home. His wife, daughters and friends saw that the act was so contrary to his natural character, that he was placed in an insane asylum, and kept there several weeks under observation for an opinion as to his responsibility. He appeared so well in the absolute quiet and rest, that he was declared sane, tried and sentenced to the state's prison, where he showed his marked mental impairment as soon as he was set to work. He could not concentrate his mind sufficiently for the simplest labor, and, a couple of years later, he was sent to the insane asylum to die—a complete mental and physical wreck, in the last stages of general paralysis."

The motor symptoms are always to be most relied upon in making a diagnosis, and especially the character of the speech, and the slight fibrillary trembling of the tongue and muscles of expression, which quiver at times as if the individual were about to burst into tears. He does not articulate plainly, especially those words abounding in consonants; he drawls, slurs his words, as if there were some difficulty in bringing them out. The muscles about the alæ of the nose, and indeed, in marked cases, all the muscles of the face, may be involved. When the tongue is protruded, it is generally with a jerk, although this symptom is usually more marked in the later stages. These motor symptoms are of grave omen, and may not be noticed if the patient be calm and collected, but they manifest themselves under excitement, and often, in examination, it is necessary to irritate the patient in order to bring them out clearly. The finer movements suffer, as seen in the handwriting, which is sometimes shaky and irregular—letters, and even words, being omitted, from inability to concentrate the mind. In some few cases, the individual, especially in the melancholic type of the disease, appears to recognize impending calamity, as the following case illustrates:

"A—— B——, thirty-four; lawyer; good family history; ambitious, and a hard worker; indulges excessively in tobacco, often smoking fifteen cigars a day; has used alcohol steadily to support strength, writes: '*Dear Sir*—I am suffering from mental and nervous prostration. I should have had advice and consultation before. My friends are much alarmed at my condition, but no more than I am. I am assured, myself, that the condition under which I am, must be controlled at once, or worse results may be apprehended.'"

In this letter, the handwriting is unsteady, and, when admitted to the asylum, his friends stated that, in writing a brief, he would frequently misspell words, or leave them out altogether, much to his annoyance, and would often have to write a letter or legal instrument over half a dozen times to secure correctness. He attempted suicide shortly before being brought to the asylum, where he died two years later from general paresis, the motor and other symptoms being all well marked.

The following case is instructive, as showing the presence of motor symptoms before the intellectual faculties became involved, and occurred in the practice of the late Dr. John P. Gray. The history was related to me in the presence of a stenographer, so I am able to give it verbatim: "A New York broker consulted me in regard to his health. He was then forty-five years of age; married; family history excellent; of rather nervous temperament; a *bon vivant,* and inclined to over-stimulation, especially when under excitement on the Exchange. He told me that he felt nervous, that he left out a word now and then in writing a letter, and that, while in the midst of a column of figures, he would lose himself and become confused. He had some tremulousness of the tongue, and, in talking to me, the muscles of his lips quivered, and there was some drawling of the speech, but it was not marked. Of this, he was not conscious. He said he wanted a candid opinion as to his condition; that there were important monied interests which it was necessary for him to attend to, and if he was going to have an attack of a serious nature, he wished to settle up his affairs. He said further, that he was not himself conscious of any mental disturbance, or that he had failed to appreciate any financial transaction. He came again the following day, and I told him that, in my opinion, he was threatened with a serious affection, and advised him to settle his affairs as soon as possible. He then explained the financial transactions to which he referred. Some weeks afterwards, he came to see me again, and the symptoms had somewhat increased, but there was no marked mental disturbance. He said he wanted to understand my opinion very distinctly again.

I told him that I now felt quite certain that he had general paresis. He closed out his business, settled all his financial affairs, and, in the course of a few months, he rather suddenly developed profound paresis, both in its mental and physical manifestations, with utter indifference with regard to it. He lived but eighteen months afterward."

General paresis may be mistaken for chronic alcoholism, but the history of the case, the difference in the character of the tremor—that of general paresis being fibrillary, and having points of election; that of chronic alcoholism being more universal, especially in the hands and arms, when outstretched—the speech, that of the general paretic, having the character already described, while that of the alcoholic subject is thick, the words being muttered; and, finally, the mental state—the contentment and indifference of the paretic—will give, in most instances, sufficient data for correct diagnosis. Acute mania, with expansive delirium, may simulate this disease, but the absence of the true physical signs will serve as a guide. Then, also, the delusions of the general paretic are not of as fixed a character as those of acute mania, a contradiction not serving to irritate the patient so much. When, however, there is, in acute mania, great emotional agitation, together with muscular tremor, due to intense excitement, time may be required to make a diagnosis with any degree of certainty. Dementia with paralysis may be mistaken for general paresis, but the apoplexy, and subsequent more or less complete paralysis, and the gradual dementia, progressive from the date of attack, will exclude general paresis. Multiple cerebro-spinal sclerosis of the descending form, intra-cranial tumor, some forms of chronic lead-poisoning, and especially diffused cerebral syphilis, may all simulate, in the early stages, incipient general paresis.

In conclusion, I would repeat that, in the diagnosis of general paresis, the motor symptoms are always most to be relied upon, and in no case should a positive opinion be hazarded without them. In practice, these are best brought out by observation of the patient when under some form of excitement, or when physically weary.

